# Dedifferentiated Carcinoma of the Ovary: A Rare Aggressive Subtype

**DOI:** 10.7759/cureus.107153

**Published:** 2026-04-16

**Authors:** Divya Bansal, Meenakshi Kamboj, Anila Sharma, Anurag Mehta

**Affiliations:** 1 Pathology, Rajiv Gandhi Cancer Institute and Research Centre, Delhi, IND; 2 Pathology and Laboratory Medicine, Rajiv Gandhi Cancer Institute and Research Centre, Delhi, IND; 3 Laboratory and Transfusion Services, Rajiv Gandhi Cancer Institute and Research Centre, Delhi, IND

**Keywords:** dedifferentiated, dedifferentiated carcinoma of ovary, endometrioid, endometrium, ovary, undifferentiated

## Abstract

Dedifferentiated carcinoma of the ovary (DDOC) is a rare and aggressive malignancy characterized by both differentiated and undifferentiated carcinoma components. While its endometrial counterpart, dedifferentiated carcinoma of the endometrium, is more frequent, DDOC remains poorly understood. We present the case of a young woman who presented with acute abdominal pain and was found to have a large complex ovarian mass. Histopathological examination revealed a combination of endometrioid carcinoma and undifferentiated carcinoma. Immunohistochemical analysis demonstrated distinct staining patterns, with the undifferentiated component exhibiting focal pan-cytokeratin positivity and loss of paired box gene 8 and estrogen receptor expression. The patient underwent standard paclitaxel-carboplatin chemotherapy but experienced rapid disease progression. DDOC poses significant diagnostic challenges due to its histological overlap with other high-grade ovarian malignancies. The presence of an undifferentiated component imparts a poor prognosis, even when it constitutes a minor portion of the tumor. Improved recognition, molecular characterization, and exploration of targeted therapies are essential for better clinical management of this rare and aggressive entity.

## Introduction

Dedifferentiated carcinoma of the ovary (DDOC) is a rare, highly aggressive neoplasm characterized by coexisting differentiated and undifferentiated carcinoma components [1]. The differentiated element is usually endometrioid carcinoma, while the undifferentiated component drives rapid progression, even when minimal [1,2]. In contrast to its endometrial counterpart (dedifferentiated carcinoma of the endometrium, DDEC), which is frequently encountered, DDOC cases have been rarely reported [1-6]. Diagnostic challenges contribute to its under-recognition, as DDOC is often misclassified as high-grade serous carcinoma (HGSC), undifferentiated carcinoma, or grade 3 endometrioid carcinoma. Immunohistochemistry (IHC) plays a critical role, with undifferentiated areas typically showing loss of paired box gene 8 (PAX8); estrogen receptor (ER), progesterone receptor (PR), variable pan-cytokeratin (panCK) staining; and frequent defective switch/sucrose non-fermentable (SWI/SNF) complex proteins, aiding distinction from mimics [1,3,7].

Proper recognition of DDOC is essential, as its clinical course, prognosis, and molecular profile differ from other ovarian tumors, necessitating distinct therapeutic considerations; HGSCs are platinum-sensitive, while DDOCs are platinum-resistant [1,8]. Here, we report a case of primary DDOC with its unique clinical, pathological, and immunohistochemical features.

## Case presentation

A 24-year-old unmarried woman presented to an outside hospital with severe lower abdominal pain. She also reported occasional intermenstrual spotting for the past two months, for which no medical consultation had been sought. Her menstrual history revealed menarche at 15 years, with regular cycles and average flow. There was no significant past, personal, or family history.

A contrast-enhanced magnetic resonance imaging of the pelvis performed elsewhere revealed a large, complex, lobulated, multiseptated, solid-cystic mass inseparable from the left ovary, measuring 18.8 × 14.9 × 8.9 cm. No fat component or calcification was identified. Post-contrast images showed inhomogeneous enhancement of the septae, solid areas, and cyst walls. The lesion extended posteriorly into the pouch of Douglas and compressed the uterus and rectum. The right ovary and endometrium appeared normal, and no significant pelvic or retroperitoneal lymphadenopathy was noted. The findings were suggestive of a left ovarian neoplasm. Serum tumor markers (cancer antigen 125, carcinoembryonic antigen, carbohydrate antigen 19-9, alpha-fetoprotein, beta-human chorionic gonadotropin, lactate dehydrogenase), along with inhibin and serum calcium levels, were within normal limits.

The patient subsequently presented to our institution. On examination, her general condition was fair, performance status was 0, and there was no abnormality in the breasts, axillae, or supraclavicular lymph nodes. Per abdomen, a large pelvic mass was palpable. Subsequently, she underwent exploratory laparotomy with staging surgery (uterine preservation performed), and a frozen section was reported as a primary malignant epithelial tumor.

**Figure 1:**
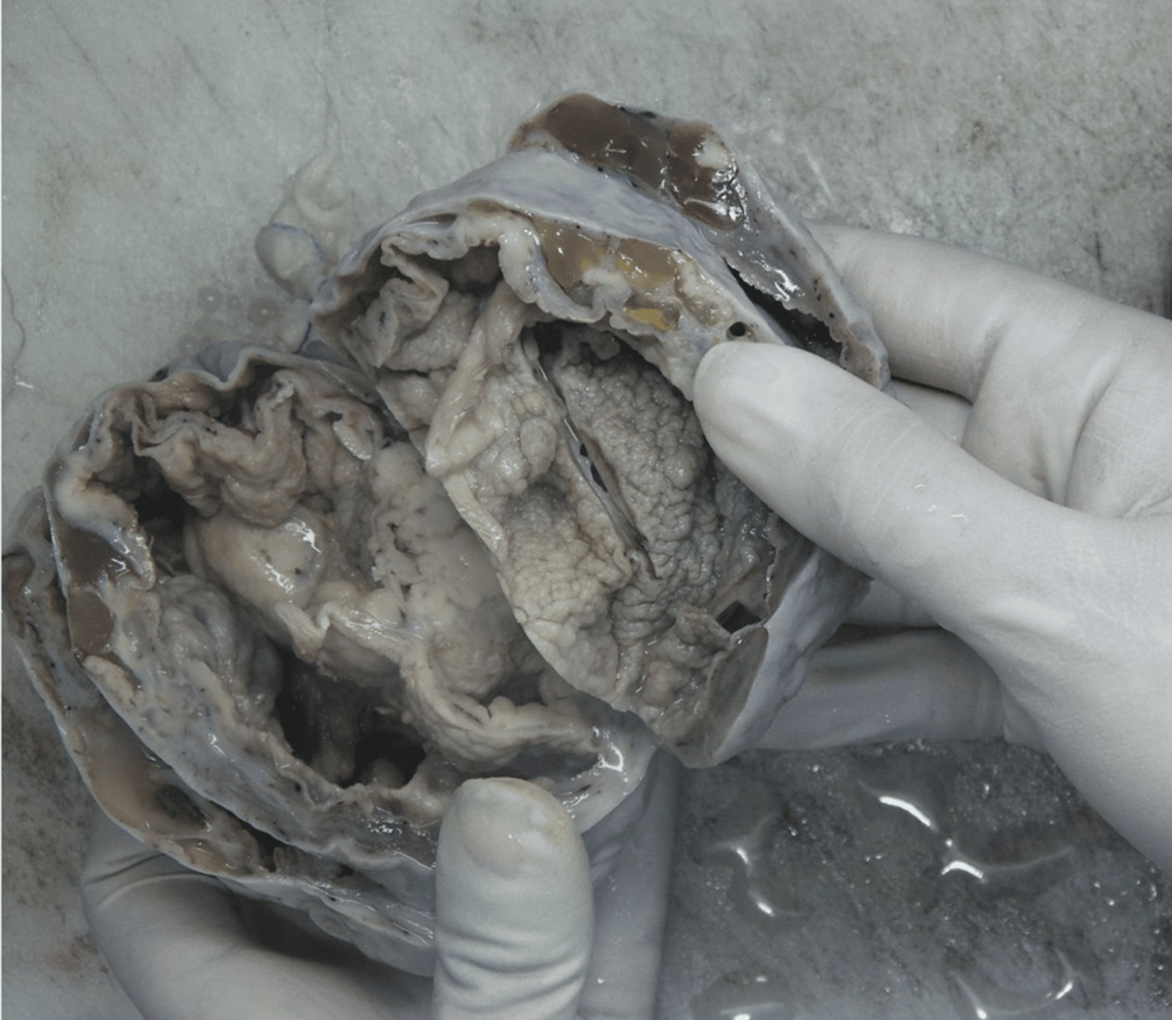
Gross image of the left adnexal mass showing a solid-cystic, gray-white tumor.

Gross examination of the left ovarian mass revealed a 19 × 13 × 7.5 cm solid-cystic, gray-white tumor with areas of necrosis and hemorrhage noted. Capsule rupture and surface involvement were also seen (Figure 1).

**Figure 2:**
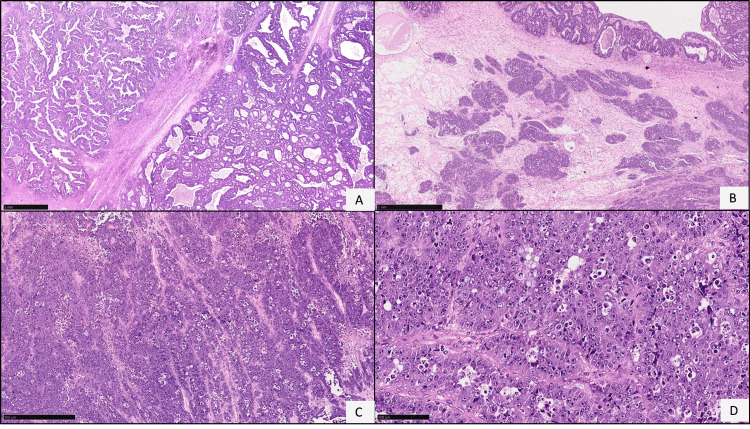
Hematoxylin and eosin images of dedifferentiated carcinoma of the ovary. (A) Endometrioid carcinoma component in a glandular pattern. (B) Areas of solid lobules were identified apart from the glandular areas. (C, D) Undifferentiated tumor morphology, tumor cells in sheets with vesicular chromatin, prominent nucleoli, brisk mitosis, and apoptosis.

Histologically, the tumor displayed a glandular and solid pattern of growth. The glandular areas showed columnar cells with vesicular chromatin and marked nuclear atypia, consistent with endometrioid adenocarcinoma, International Federation of Gynecology and Obstetrics (FIGO) Grade 2 (Figure 2A). The solid areas had a sheet-like growth with tumor cells exhibiting vesicular chromatin, prominent nucleoli, brisk mitosis, apoptosis, and necrosis, indicative of undifferentiated carcinoma (Figures 2B-2D).

Extensive lymphovascular emboli and metastatic deposits in the left pelvic and retroperitoneal lymph nodes, as well as posterior peritoneal involvement, were noted. The cystic component showed endometriosis, while the bilateral fallopian tubes and the right ovary were free of tumor.

**Figure 3:**
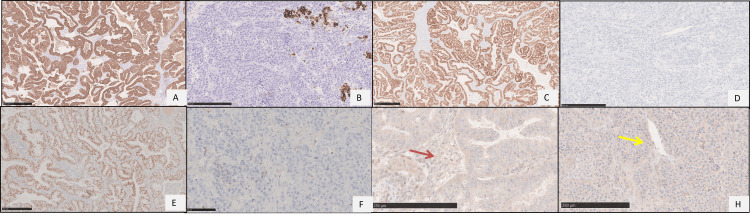
Immunohistochemical images of dedifferentiated carcinoma of the ovary. A, C, E, G: differentiated component; B, D, F, H: undifferentiated component. (A) Pan-cytokeratin (Pan-CK) diffuse positivity in endometrioid carcinoma. (B) Pan-CK focal positivity in undifferentiated carcinoma component. (C) Paired box gene 8 (PAX8) diffuse positivity in endometrioid carcinoma. (D) PAX8 negative in undifferentiated carcinoma component. (E) Estrogen receptor (ER) diffuse positivity in endometrioid carcinoma. (F) ER negative in undifferentiated carcinoma component. (G, H) p53 null pattern of immunoexpression (mutant type) in both endometrioid (G) and undifferentiated carcinoma component (H). Note, the wild pattern of staining in the internal control, i.e., stromal cells (red arrow, G; yellow arrow, H).

On IHC, glandular areas expressed pan-cytokeratin (pan-CK), cytokeratin 7 (CK7), PAX8, and ER, while the solid areas showed focal pan-CK and CK7 positivity but were negative for PAX8 and ER. Both components demonstrated a null (mutant-type) p53 immunoexpression pattern with intact Brahma-related gene 1 (BRG1) and integrase interactor 1 (INI1) and negative Wilms tumor gene 1 (WT1), Spalt-like transcription factor 4 (SALL4), synaptophysin, and INSM transcriptional repressor 1 (INSM1) (Figures 3A-3H). A final diagnosis of DDOC was rendered, with glandular areas representing endometrioid carcinoma and solid areas representing undifferentiated carcinoma.

Although the uterus was preserved, a thorough evaluation was performed to rule out a synchronous endometrial neoplasm. Preoperative imaging demonstrated a normal endometrium. Intraoperatively, the uterus was inspected and palpated, revealing no abnormalities. The presence of endometriosis in the ovarian cystic component further supported a primary ovarian origin, as it provides a background from which ovarian endometrioid carcinoma can arise.

**Figure 4:**
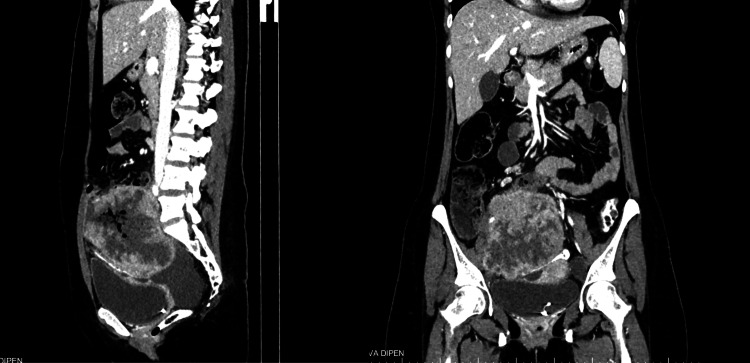
Sagittal and coronal contrast-enhanced CT scan showing a large well-defined heterogeneously enhancing necrotic right adnexal mass.

The case was discussed in a multidisciplinary tumor board meeting (MTB). In the absence of established guidelines for DDOC, the patient was treated with standard paclitaxel-carboplatin, as used for high-grade epithelial ovarian cancers. Although emerging evidence suggests relative platinum resistance, particularly in the undifferentiated component, this regimen remains a pragmatic first-line option given the lack of validated alternatives and the patient’s good performance status. The patient received paclitaxel at a dose of 175 mg/m² and carboplatin at an area under the curve of 5-6, administered every three weeks for six cycles. Mismatch repair (MMR) testing for MMR proteins (MLH1, PMS2, MSH2, MSH6) by IHC was performed and showed intact nuclear immunoexpression of all MMR proteins and, thus, MMR proficient. Somatic *BRCA1/2* testing revealed no clinically significant mutations. At follow-up four months after completion of chemotherapy, an ultrasound of the abdomen showed a right pelvic lesion. Subsequent CT of the abdomen also revealed a large heterogeneously enhancing necrotic soft-tissue density mass right adnexal lesion along the right internal and external iliac region measuring 76 × 56 mm and abutting the right internal and external iliac vessels. It was infiltrating the right psoas major muscles (Figure 4).

Subsequently, pelvic mass biopsy showed features consistent with undifferentiated carcinoma, confirming disease progression. After the MTB discussion, given progression within six months of platinum therapy, the disease was classified as platinum-refractory, and second-line treatment with liposomal doxorubicin was initiated in line with standard approaches for platinum-resistant ovarian cancer.

During the second cycle of liposomal doxorubicin, the patient developed acute hypotension and tachycardia, necessitating transfer to the intensive care unit. Given the close temporal association with chemotherapy, several possibilities were considered, including treatment-related cardiotoxicity (though less likely with liposomal formulations), sepsis in the setting of possible neutropenia, and rapid disease progression with systemic compromise.

Laboratory workup showed leucocytosis, elevated inflammatory markers, and no evidence of cardiac enzyme elevation. Echocardiography did not reveal significant left ventricular dysfunction. Despite evaluation, a single definitive cause could not be identified, and the clinical picture was likely multifactorial in the context of aggressive disease.

The patient was subsequently transitioned to best supportive care and passed away shortly thereafter. In view of the rapid progression, early platinum resistance, and deterioration despite second-line therapy, the death was considered most consistent with disease progression rather than treatment-related toxicity.

## Discussion

DDOC and undifferentiated ovarian carcinoma are rare and aggressive entities that have not been well characterized in the existing literature [1-3,5,6]. A study by Tessier-Cloutier et al. represents the largest clinicopathologic and molecular analysis of these 30 tumors to date [1]. In their study, the age range was wide (22-71 years, median = 55 years), and the majority of the patients presented at an advanced stage; however, our patient was a young woman. Histologically, the differentiated component of the 20 DDOC displayed pure classic endometrioid histology in 17 cases, with characteristic immunohistochemical features with loss of PAX8 and ER in undifferentiated areas, and variable cytokeratin expression in their study. Loss of SMARCA4 (BRG1) was seen in approximately 40% of cases, and MMR deficiency was observed in about 25% of cases. These tumors exhibited poor response to platinum-based chemotherapy and carried a dismal prognosis even when the undifferentiated component was minor (<5%) [1].

DDOC can present with challenges in differential diagnosis, particularly in biopsy samples limited to the undifferentiated component. This undifferentiated component can have variable morphology ranging from more dyscohesive to rhabdoid to a solid nested pattern, as seen in our case, which had a predominantly solid pattern [1]. Extensive sampling on a resection specimen, including a cystic component, can identify differentiated areas. The solid areas in combination with glandular areas raise a differential diagnosis of endometrioid carcinoma, FIGO grade 3, HGSC, or composite tumor with endometrioid and neuroendocrine carcinomas (NEC). Hence, an extensive but judicious use of IHC markers is crucial for making a diagnosis of DDOC. The pattern of immunoexpression (loss or very focal) of panCK, PAX8, and ER, along with morphology, suggests an undifferentiated component. WT1 and synaptophysin negativity, along with this distinct IHC pattern in the undifferentiated component, rule out HGSC and NEC, respectively, as seen in our case. Moreover, a significant proportion of DDOCs are associated with synchronous low-grade endometrioid carcinomas of the endometrium, often representing primary endometrial neoplasms with ovarian metastasis [2,4,5]. However, in our case, a thorough evaluation revealed no synchronous endometrial component. The presence of endometriosis within the ovarian cystic component further supports a primary ovarian origin, as this is a well-recognized precursor lesion for ovarian endometrioid carcinoma [9].

Molecular alterations in DDOC commonly involve the inactivation of ARID1A, ARID1B, SMARCA4, and SMARCB1, key components of the SWI/SNF chromatin remodeling complex [1-3]. DNA MMR deficiency is observed in about 25% of DDOC cases, less frequently than in DDEC (50%-80%) [1]. IHC for SWI/SNF and MMR proteins aids in diagnosis. However, our case did not show any BRG1/INI1 loss, and MMR was proficient. The p53 null pattern observed in this case is less common in DDOC, with most reported cases showing either wild-type p53 expression or, less frequently, aberrant overexpression [1]. The significance of this finding in DDOC remains to be fully elucidated, but it may represent an alternative molecular pathway contributing to tumor aggressiveness, particularly in the context of retained SWI/SNF complex proteins.

Due to its rarity, there are no standardized treatment protocols for DDOC. Standard chemotherapy for ovarian carcinomas, platinum-taxane-based regimens (e.g., carboplatin and paclitaxel), is commonly used. However, dedifferentiated components exhibit platinum resistance, resulting in poor treatment response [1,8]. The prognostic significance of the undifferentiated component is such that its presence, even when constituting as little as 1% of the tumor, has been associated with a higher risk of recurrence and poorer overall survival compared to pure endometrioid carcinomas [1,5]. Hence, extensive sampling of the specimens is critical. In our case, the undifferentiated component constituted a significant proportion of the tumor, consistent with the aggressive clinical course characterized by rapid progression and platinum resistance. The undifferentiated component often exhibits stem-like properties and upregulation of DNA repair pathways, which may contribute to chemoresistance. The rapid progression following platinum-based therapy in our patient underscores the need for alternative treatment strategies.

Whole-genome sequencing and molecular profiling may help identify actionable mutations for individualized therapy. Clinical trials evaluating alternative treatment strategies [8], including immune checkpoint inhibitors [10], EZH2 inhibitors [11,12], and combination therapies [10], are warranted to improve outcomes for patients with this challenging malignancy.

## Conclusions

DDOC is an exceedingly rare and aggressive malignancy that poses significant diagnostic and therapeutic challenges. Its distinction from other high-grade ovarian tumors is critical, as the presence of an undifferentiated component is associated with poor prognosis and chemoresistance, underscoring the need for novel therapeutic approaches. Heightened awareness and the strategic use of IHC in poorly differentiated tumors are essential for improving diagnostic accuracy and patient management in this rare and aggressive malignancy. Future studies focusing on molecular characterization and targeted therapies, such as immune checkpoint inhibitors and epigenetic modifiers, may provide more effective treatment options for DDOC.
